# Ascertaining the relationship between *Salmonella* Typhimurium and *Salmonella* 4,[5],12:i:- by MLVA and inferring the sources of human salmonellosis due to the two serovars in Italy

**DOI:** 10.3389/fmicb.2015.00301

**Published:** 2015-04-27

**Authors:** Lisa Barco, Federica Barrucci, Enzo Cortini, Elena Ramon, John E. Olsen, Ida Luzzi, Antonia A. Lettini, Antonia Ricci

**Affiliations:** ^1^Food Safety Department, OIE and National Reference Laboratory for Salmonella, Istituto Zooprofilattico Sperimentale delle Venezie, LegnaroItaly; ^2^Department of Veterinary Disease Biology, Faculty of Health and Medical Sciences, University of Copenhagen, CopenhagenDenmark; ^3^Department of Infectious, Parasitic and Immune-Mediated Diseases, Istituto Superiore di Sanità, RomeItaly

**Keywords:** *Salmonella*, genetic similarities, microbial subtyping, source attribution, MLVA

## Abstract

The current picture of human salmonellosis shows *Salmonella* Typhimurium and *S*. 4,[5],12:i:- as the most common serovars in Italy. The aims of this study were to investigate the genetic relationship between these serovars, as well as to test the possibility of inferring sources of human salmonellosis due to *S*. Typhimurium and *S*. 4,[5],12:i:- by using multilocus variable-number tandem repeat analysis (MLVA) subtyping data. Single isolates from 268 human sporadic cases and 325 veterinary isolates (from pig, cattle, chicken, and turkey) collected over the period 2009–2011 were typed by MLVA, and the similarities of MLVA profiles were investigated using different analytical approaches. Results showed that isolates of *S*. 4,[5],12:i:- were more clonal compared to *S*. Typhimurium and that clones of both serovars from different non-human sources were very close to those which were responsible for human infections, suggesting that source attribution by MLVA typing should be possible. However, using the Asymmetric Island Model it was not possible to obtain a confident ranking of sources responsible for human infections based on MLVA profiles. The source assignments provided by the model could have been jeopardized by the high heterogeneity found within each source and the negligible divergence between sources as well as by the limited source data available, especially for some species.

## Introduction

*Salmonella* is the second most frequent zoonosis in the European Union. In 2012, the top two *Salmonella* serovars isolated from humans were *S*. Enteritidis (41.4%) and *S*. Typhimurium (22.1%); (European Food Safety Authority [EFSA], and European Centre for Disease Prevention, and Control [ECDC], 2014). In Italy, contrary to the majority of the European countries, *S*. Typhimurium has been the most common serovar since 2000 (Graziani et al., 2013). Recently, another serovar, *S*. 4,[5],12:i:-, has sharply increased in prevalence. This serovar was isolated for the first time from humans in Italy in 2003. Since then a constant and progressive increase has been observed, and *S*. 4,[5],12:i:- accounted for almost 40% of human isolates in 2011 (Dionisi et al., 2009). Similar trends have been observed in other parts of Europe (European Food Safety Authority [EFSA], 2010; Hopkins et al., 2010), and in a situation where *Salmonella* isolations in general have been progressively decreasing, *S*. 4,[5],12:i:- is one of the few serovars for which an opposite trend has been described (European Food Safety Authority [EFSA], and European Centre for Disease Prevention, and Control [ECDC], 2014). *S*. 4,[5],12:i:- is defined as a monophasic variant of *S*. Typhimurium (4,[5],12:i:- 1,2) because of antigenic and genetic similarities between the two serovars, and the characterization of *S*. 4,[5],12:i:- isolates by using different molecular approaches demonstrated that *S*. Typhimurium is the direct ancestor of *S*. 4,[5],12:i:- (Soyer et al., 2009).

Multilocus variable-number tandem repeat analysis (MLVA) has been increasingly used in Europe as a primary method for *S.* Typhimurium subtyping especially in the context of outbreak investigations (Torpdahl et al., 2007; Petersen et al., 2011; Ross et al., 2011). A 5-loci MLVA scheme (Lindstedt et al., 2004) has been standardized and recently validated in a large European inter-laboratory trial (Larsson et al., 2013). MLVA has been identified as one of the most valuable subtyping methods for *Salmonella* (European Food Safety Authority [EFSA], 2008, 2013; Barco et al., 2013), mainly thanks to the possibility of automation, which facilitates the analysis of a large number of isolates. Another strength of MLVA is the output it produces. A MLVA profile consists of a string of numbers, which is easily shared among laboratories and suitable to supply mathematical models (Wuyts et al., 2013).

In order to correctly allocate the available resources to prevent human foodborne diseases, it is important for risk managers to be able to accurately apportion sporadic cases of infection to specific animal hosts and to understand transmission routes of the pathogens (Havelaar et al., 2007; Heck, 2009). Efforts to quantify the importance of specific sources and animal reservoirs responsible for human infection have been gathered under the term source attribution (European Food Safety Authority [EFSA], 2008), which has been defined as the partitioning of the human disease burden of one foodborne pathogen to specific sources, whether being animal reservoirs or vehicles for transmission through the food chain (Pires et al., 2009). Even though different source attribution approaches have been described, microbial subtyping source attribution methodology has been the most frequently used (see review by Barco et al., 2013). The principle behind this methodology is the comparison of the subtypes in putative sources with the subtypes identified in human samples (Pires et al., 2009). This methodology requires a collection of temporally and spatially related isolates from different sources and from humans (European Food Safety Authority [EFSA], 2008). The great majority of *Salmonella* source attribution exercises carried out so far have been based on frequency-matching models, which compare the distribution of subtypes identified in humans with those in the putative sources in order to infer the principal sources of human infections (European Food Safety Authority [EFSA], 2013). These models have been implemented by using phenotypic subtyping data (e.g., serovars, phage-type, and antimicrobial resistance profiles). As an alternative models that consider the population genetics of foodborne bacteria can be used. Mathematical models, which estimate the amount of mutations, recombination, and migrations of the target DNA from different sources, can be valuable tools to probabilistically assign human cases to the putative sources (European Food Safety Authority [EFSA], 2013). The Asymmetric Island Model is an example of a source attribution model that uses this principle. It was originally applied to estimate sources of human campylobacteriosis based on multilocus sequence typing (MLST) data (Wilson et al., 2008). More recently, a Dutch study took advantage of this model to estimate the main sources of human salmonellosis due to *S*. Typhimurium, its monophasic variant and *S*. Enteritidis based on MLVA profiles (Mughini-Gras et al., 2014a).

Although different studies have demonstrated that the monophasic serovar emerged from *S*. Typhimurium through multiple independent emergence events (Soyer et al., 2009; Switt et al., 2009), the genetic relationship between the two serovars deserves further investigations, in order to collect valuable information to explore the reasons for the sharp emergence of *S*. 4,[5],12:i:- isolates and find plausible explanations for its evolutionary success.

The aims of the present study were (i) to investigate the relationship between *S*. Typhimurium and *S*. 4,[5],12:i:-, as the most important serovars circulating in Italy, and (ii) to test the possibility of inferring sources of human salmonellosis due to these two serovars by using MLVA subtyping data.

## Materials and Methods

### Data Set

Single isolates from 268 human sporadic cases and 325 veterinary isolates of *S*. Typhimurium and *S*. 4,[5],12:i:- (**Table 1**) were collected in Italy between January 2009 and December 2011. The isolates were epidemiologically unrelated to the extent that could be established. Human cases were identified through “Enter-net Italia,” a passive laboratory-based surveillance system for *Salmonella* based on the contribution of 140 peripheral laboratories under the supervision of the Istituto Superiore di Sanità (Rome). Veterinary isolates were collected in the framework of the “Enter-vet” network, a laboratory surveillance system in place in Italy for the collection of veterinary isolates of *Salmonella*. This network consists of 10 peripheral laboratories distributed throughout the Country and it is coordinated by the Italian National Reference Laboratory for *Salmonella* (Istituto Zooprofilattico Sperimentale delle Venezie, Legnaro, Padova). Veterinary isolates were collected both at reservoir level (from animal samples) and at the point of purchase and consumption (from food samples), in order to trace the putative sources along the entire food production chain. Poultry samples were obtained at primary production level in the context of EU control programs (according to Regulation EC No 2160/2003) and for other species in the context of locally implemented programs, as well as from *ad hoc* surveillance activities following suspected incidents. Food samples were collected both at the production and retail levels according to the control programs implemented to verify the correct application of Regulation EC No 2073/2005. The number of non-human isolates were distributed among the sources according to the frequencies reported for *S.* 4,[5],12:i:- and *S.* Typhimurium within the Enter-vet network during the considered time frame.

**Table 1:** Distribution of isolates by serovar and source.

Serovar	Source	N^∘^ of isolates	N^∘^ of MLVA profiles	N^∘^ isolates with unshared MLVA profile (%)^a^
*S.* Typhimurium	Human	63	28	28 (44.4)
	Pig	73	44	47 (64.4)
	Chicken	20	15	17 (65)
	Cattle	18	15	11 (61.1)
	Turkey	8	6	3 (37.5)
	Total	182	93	106 (58.2)

*S*. 4,[5],12:i:-	Human	205	53	38 (18.5)
	Pig	131	43	26 (19.8)
	Chicken	27	17	1 (3.7)
	Cattle	36	12	1 (2.8)
	Turkey	12	10	2 (16.7)
	Total	411	79	68 (16.5)

*Number of different MLVA profiles identified per each source; number of isolates and percentage () of MLVA profiles identified only in one specific source.*

*^a^The word unshared here refers to isolates belonging to a MLVA profile type only seen among human isolates or among just one of the sources investigated.*

### Analytical Methods

#### Serotyping and PCR Confirmation Test

*Salmonella* 4,[5],12:i:- and *S.* Typhimurium isolates were serotyped by slide agglutination with commercial antisera according to the White-Kauffmann-Le Minor scheme (Grimont and Weill, 2007). Moreover, in order to ascertain the monophasic or biphasic status of the isolates the PCR protocol recommended by the European Food Safety Authority (European Food Safety Authority [EFSA], 2010; Barco et al., 2011) was used. This multiplex PCR protocol allows the simultaneous amplification of the phase-2 flagellar gene (*flj*B), which is detected only among the biphasic isolates and the *fliA-B* intergenic region, generating a 1 kb amplicon that is specific for *S.* 4,[5],12:i:- and *S.* Typhimurium and that is due to the presence of an IS*200* copy.

#### Multilocus Variable-Number Tandem Repeat Analysis

Multilocus variable-number tandem repeat analysis was performed according to the protocol described by Lindstedt et al. (2004). The size measurements for each locus were estimated using a Genetic Analyzer 3130XL (Applied Biosystems, Life Technologies Corporation, Carlsbad, CA, USA). A set of 33 reference *S*. Typhimurium isolates (provided by the Statens Serum Institut, Copenhagen, Denmark) were used to normalize the raw data obtained from the analysis of all isolates by capillary electrophoresis using GeneMapper (software version 4.0, Applied Biosystems Science, Life Technologies Corporation). According to the nomenclature suggested by Larsson et al. (2009), MLVA results were reported as a string of five numbers representing the variable number of tandem repeats (VNTRs) at the corresponding loci (STTR9–STTR5–STTR6–STTR10pl–STTR3), or as 0 in the case that a PCR product was not obtained for a locus. VNTR allele numbers were imported as character values into the BioNumerics Software (version 6.6, Applied Maths, NV, Saint-Martens — Latem, Belgium) for analysis, then subjected to cluster analysis and dendrogram construction by the unweighted pair-group method using arithmetic averages (UPGMAs) clustering, using a distance measure based on the number of different loci between profiles. To visualize the relationships between isolates, standard minimum spanning trees (MSTs) were generated using categorical coefficient, the single and double locus variance priority rules and avoiding the creation of hypothetical types. Clonal complexes were created based on maximum neighbor distance of changes at two loci and a minimum of two MLVA profiles per complex.

### Statistical Analyses

#### Descriptive Analyses

A descriptive analysis of MLVA profile frequencies in the two serotypes and VNTR loci variability between human and non-human sources was conducted by using the R version 3.1.2 (R Core Team, 2012).

#### Diversity Index

The diversity among the five VNTR (STTR9–STTR5–STTR6–STTR10pl–STTR3) was estimated according to the Simpson’s Diversity Index, which quantifies the variation of the number of repeats at each locus and assumes values ranging from 0.0 (indicative of complete absence of diversity) to 1.0 (indicative of complete diversity). To calculate the index, the online toll “DIversity and Confidence Extractor (V-DICE)” provided by the Health protection Agency’s Bioinformatics Unit (available at http://www.hpa-bioinformatics.org.uk/cgi-bin/DICI/DICI.pl) was used.

#### Asymmetric Island Model

The Asymmetric Island Model was applied as described originally by Wilson et al. (2008). The model is an evolutionary model assuming that the *Salmonella* population consists of a number of discrete islands, each of which corresponds to a different source, and allowing for occasional exchange between islands (migrations), generation of new MLVA profiles (mutation) and recombination. Formally, the model is a Bayesian model in two stages: in the first stage the model estimates the posterior distributions of the evolutionary parameters (migration, mutation and recombination), based on source data, and in the second stage the estimated posterior distributions are used in order to infer the fraction of human cases attributable to each source.

The model was run considering *S*. Typhimurium and *S*. 4,[5],12:i:- MLVA profiles separately as well as considering a unique dataset (merged database) including all MLVA profiles irrespective of the serovar. Since few MLVA typed isolates were available for chickens and turkeys, these isolates were pooled so that the attribution was performed considering the “poultry” source.

Moreover, in order to assess the sensitivity of the model to the sample size differences between sources, bootstrap samples of equal size were constructed for each source by sampling 100 times with replacement from the original sample. Also this new dataset, consisting of the original data for human isolates and the bootstrap samples for the source isolates, was used to run the model.

#### Analysis of Molecular Variance

To quantify genetic differentiation between the different populations investigated (human and putative sources), analysis of molecular variance (AMOVA) was used. AMOVA explicitly extends the procedures and formats used in the traditional analysis of variance, in order to estimate the degree of genetic differentiation between-group and within-group at several hierarchical levels (Excoffier et al., 1992). AMOVA produces the variance components of each hierarchical level and estimates the Phi statistic, the commonly used index that represents the distribution of allelic diversity across multiple levels of population subdivision. A higher value for the Phi statistic represents a higher amount of population differentiation. AMOVA was performed by partitioning the datasets into a hierarchical structure. At the top there were “regions” including human and non-human isolates, then at the second level there were “populations” including human isolates and isolates from the different sources separately and at the third level there were MLVA profiles associated with each isolate. The Phi statistic was calculated at the following levels: (i) between human and non-human isolates, (ii) between each source, (iii) within each source. The AMOVA analysis was conducted by using the R package ade4 (Dray and Dufour, 2007) and using the Euclidean distance to construct the distance matrix.

## Results

### Comparison of MLVA Profiles between *S.* Typhimurium and S. 4,[5],12:i:- Isolates

Although in the dataset contained more isolates of *S.* 4,[5],12:i:- than *S*. Typhimurium (411 and 182 isolates respectively), the number of MLVA profiles associated with the first serovar (93) was higher than the number associated with the latter one (79; **Table 1**). 25 MLVA profiles were shared by the two serovars, accounting for 55.49 and 75.91% of *S*. Typhimurium and *S.* 4,[5],12:i:- isolates respectively.

**FIGURE 1:**
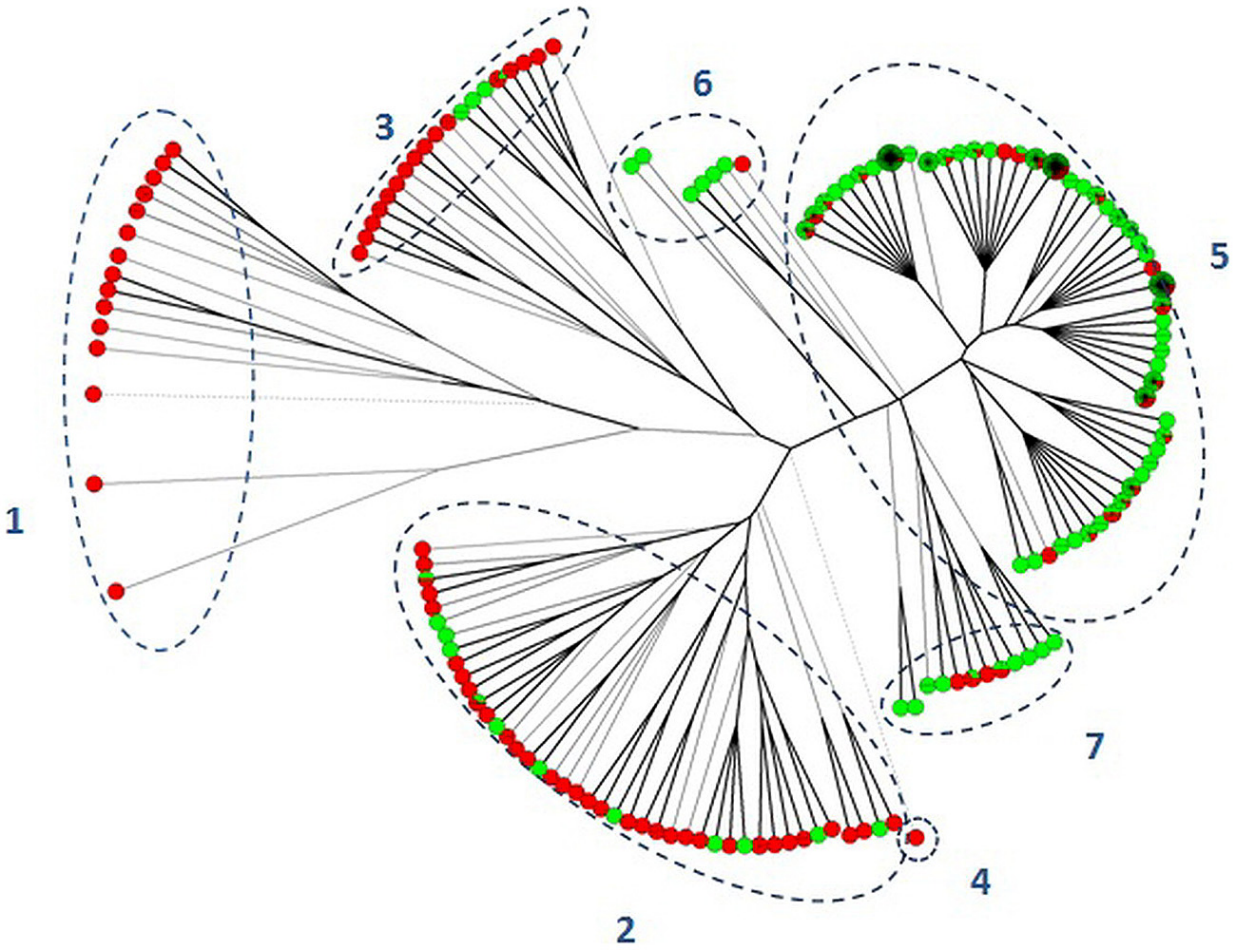
**Unweighted pair-group method using arithmetic average clustering of *Salmonella* Typhimurium (red balls) and *S*. 4,[5],12:i:- (green balls) isolates.** Dotted lines separated isolates into different clusters (1–7).

Cluster analysis by UPGMA was performed to clarify the relationship between the two serovars (**Figure 1**). The entire dataset was distributed into seven different clusters. Two clusters included MLVA profiles exclusively associated with *S.* Typhimurium (clusters 1 and 4). The remaining five clusters showed MLVA profiles associated with both serovars. Within clusters 2 and 3, isolates of *S.* Typhimurium were more common in comparison to profiles associated with the monophasic variant, while profiles associated with *S.* 4,[5],12:i:- were more common within clusters 5, 6, and 7. Cluster analysis confirmed that some profiles remain specifically associated with one of the two serovars, and some degree of differentiation between the two serovars occurs.

The degree of polymorphism of MLVA profiles associated with the two serovars was quantified by calculating the diversity index (**Table 2**) for the five VNTR included in the *S*. Typhimurium MLVA scheme. For *S.* Typhimurium, the diversity index ranged from 0.37 (STTR9) to 0.87 (STTR6). The most diverse loci were STTR6 and STTR5, which generated 18 and 17 alleles respectively. STTR10 generated 19 alleles, but the final diversity index was lower compared to STTR6 and 5, since for 62% of the *S*. Typhmurium isolates, amplification at locus STTR10 was not generated. For the last two loci (STTR3 and 9) the diversity indexes were equal to 0.51 and 0.37 respectively.

**Table 2:** Simpson’s index of diversity for the five VNTR of the MLVA scheme estimated for *S*. Typhimurium and *S*. 4,[5],12:i:-.

	*S*. Typhimurium				*S*. 4,[5],12:i:-			
Locus	Diversity index	Confidence interval^∗^	K^#^	Max (pi)^+^	Diversity index	Confidence interval^∗^	K^#^	Max (pi)^+^
STTR6	0.87	0.84–0.89	18	0.26	0.78	0.75–0.80	14	0.34
STTR5	0.82	0.79–0.86	17	0.34	0.72	0.70–0.75	11	0.40
STTR10	0.60	0.52–0.69	19	0.62	0.06	0.03–0.09	7	0.97
STTR3	0.51	0.44–0.58	10	0.66	0.13	0.09–0.18	8	0.93
STTR9	0.37	0.28–0.45	4	0.78	0.02	0.00–0.04	2	0.10

*^∗^Precision of the Diversity Index, expressed as 95% upper and lower boundaries.*

*^#^Number of different repeats present at this locus.*

*^+^Fraction of isolates that have the most frequent repeat number in this locus (range: 0.0–1.0).*

For *S*. 4,5,12:i:- only, two out of five loci were polymorphic. For STTR6 and 5, which generated 14 and 11 alleles respectively, the diversity indexes were equal to 0.78 and 0.72. The remaining three loci, STTR3, 10 and 9 had a lack of discrimination. Their diversity indexes were equal to 0.13 (STTR3), 0.06 (STTR10) and 0.02 (STTR9), indicative of insignificant polymorphism.

### Source Attribution

#### Descriptive Analysis

The unshared MLVA profiles, defined as profiles that were exclusively displayed by human isolates or by one specific source, comprised 58.2 and 16.5% of the total number of *S*. Typhimurium and *S.* 4,[5],12:i:- isolates, respectively (**Table 1**). With regard to *S*. Typhimurium, six out of 28 human MLVA profiles identified (accounting for 55.5% of all human isolates) were also found among isolates from one of the investigated sources. One MLVA profile was found among human isolates as well as three different sources (pig, chicken, and cattle), one human MLVA profile was shared by two different sources (pig and cattle) and the remaining four human MLVA profiles were only recovered from pig isolates. All shared human MLVA profiles associated with *S*. Typhimurium were found also in swine isolates (**Table 3**).

**Table 3:** List of MLVA profiles for *S*. Typhimurium and *S.* 4,[5],12:i:- shared by human and non-human sources.

	MLVA profile	N^∘^ of isolates				
		Human	Pig	Chicken	Cattle	Turkey
*S*. Typhimurium	3_12_10_0_211	2	4	2	3	0
	3_13_11_0_211	3	2	0	1	0
	3_12_11_0_211	7	3	0	0	0
	3_12_9_0_211	20	1	0	0	0
	3_15_11_0_311	1	3	0	0	0
	4_13_9_7_211	2	2	0	0	0

*S.* 4,[5],12:i:-	3_12_10_0_211	21	16	4	7	2
	3_12_9_0_211	23	17	2	5	1
	3_12_7_0_211	1	3	1	2	1
	3_13_9_0_211	8	7	1	3	1
	3_11_8_0_211	8	1	2	2	0
	3_11_9_0_211	6	8	1	7	0
	3_12_11_0_211	8	8	2	1	0
	3_13_10_0_211	49	11	2	3	0
	3_12_8_0_211	5	8	4	1	0
	3_13_11_0_211	5	1	1	1	0
	3_12_12_0_211	3	2	1	0	1
	3_13_8_0_211	6	4	1	0	1
	3_10_10_0_211	3	3	1	0	0
	3_11_10_0_211	3	2	1	0	0
	3_11_11_0_211	1	2	0	0	1
	3_15_9_0_211	1	3	0	0	0
	3_11_12_0_211	3	0	0	0	1
	3_11_13_0_211	1	1	0	0	0
	3_13_7_0_211	2	2	0	0	0
	3_14_10_0_211	8	1	0	0	0
	3_14_11_0_211	2	2	0	0	0

With regard to *S.* 4,[5],12:i:-, 21 out of 53 human MLVA profiles detected (accounting for 81.47% of all human isolates) were also displayed by isolates from other sources. Four human MLVA profiles were also found in isolates from all the investigated sources, eight human MLVA profiles were shared by isolates from three different sources (pig, chicken, and cattle for six profiles; pig, chicken, and turkey for two profiles); three human MLVA profiles were shared by two different sources (pig and chicken for two profiles; pig and turkey for the third profile) and the remaining six human MLVA profiles were detected in only one source (pig in five cases and turkey in the last case). All but one of the human shared MLVA profiles were also displayed by pig isolates (**Table 3**).

#### Minimum Spanning Tree

**FIGURE 2:**
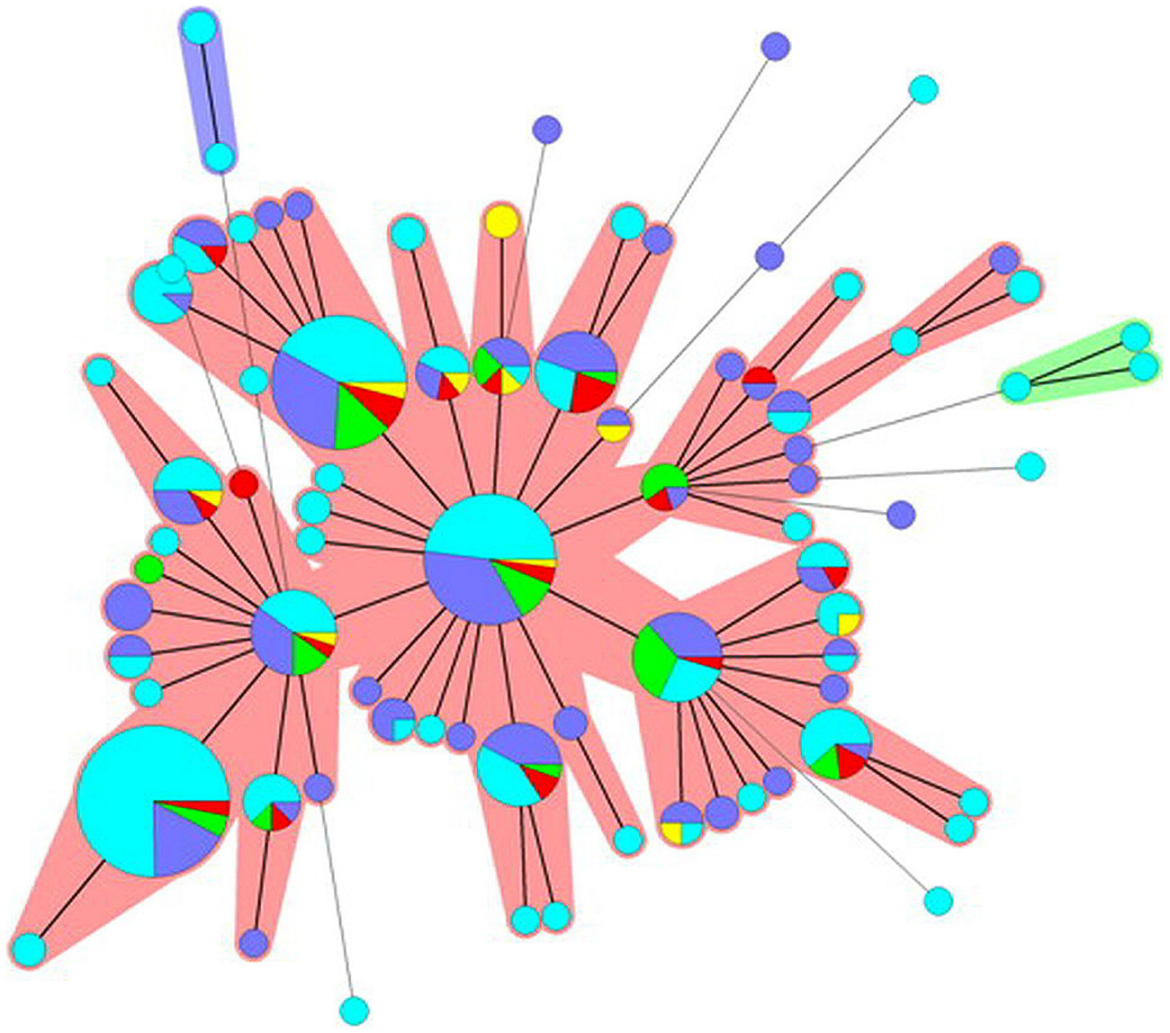
**Minimum spanning tree based on the MLVA profiles observed for *S*. Typhimurium isolates.** Each node corresponds to a MLVA profile and each node size is proportional to the number of isolates displaying this particular profile. The length and thickness of the branches are proportional to the number of the loci differing between two profiles. The color code used reflects the origin of the isolates: light blue, human; purple, pig; green, cattle; red, chicken; yellow, turkey. Halos indicate different clonal complexes.

**FIGURE 3:**
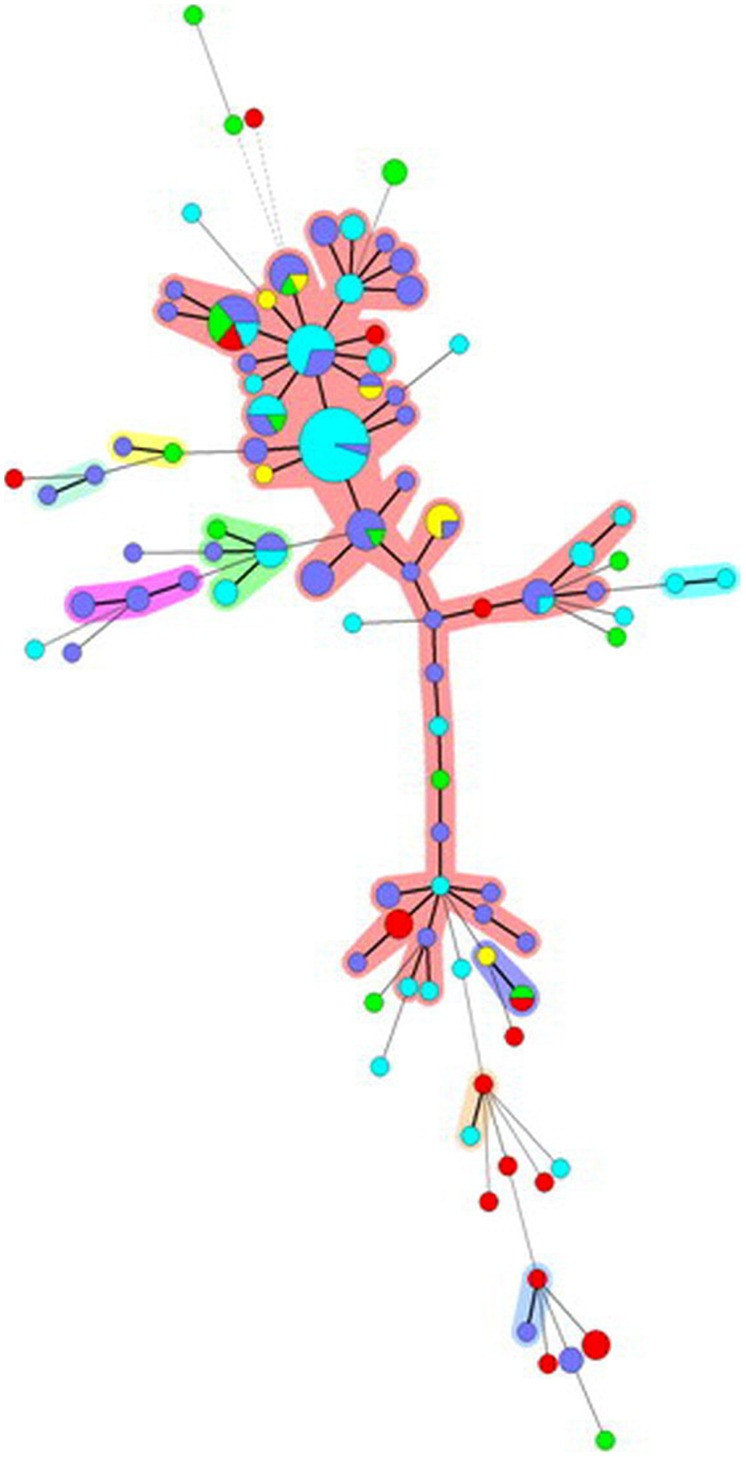
**Minimum spanning tree based on the MLVA profiles observed for *S*. 4,[5]:i:- isolates.** Each node corresponds to a MLVA profile and each node size is proportional to the number of isolates displaying this particular profile. The length and thickness of the branches are proportional to the number of the loci differing between two profiles. The color code used reflects the origin of the isolates: light blue, human; purple, pig; green, cattle; red, chicken; yellow, turkey. Halos indicate different clonal complexes.

Cluster analysis based on similarities of MLVA profiles using MST for *S*. Typhimurium showed one major and eight minor clusters (including from 2 to 4 different MLVA profiles). The major cluster included MLVA profiles associated with human isolates as well as isolates from different sources, even though very few MLVA profiles were shared between human and non-human isolates (**Figure 2**). For *S*. 4,[5]12:i:- the picture obtained was different since the MST consisted of only one major and two minor clusters (**Figure 3**). The minor clusters included few MLVA profiles displayed by human isolates, whereas within the major cluster, the most common MLVA profiles were shared by human isolates and by isolates from all the sources, indicating multiple contamination sources for monophasic isolates responsible for human infections.

#### Asymmetric Island Model

The Asymmetric Island model attributed most human cases to the pig sources for both serovars (64.4%, 95% credibility interval [95% CI] 27.7–89.3% for *S*. Typhimurium and 58.4%, 95% CI 1.8–96.2% for *S*. 4,[5],12:i:-). Cattle were identified as the second most important source for *S*. Typhimurium (21.5%, 95% CI 0.5–63.8%) in humans followed by poultry (14.1%, 95% CI 0.3–45.6%). With regard to *S*. 4,[5],12:i:-, poultry was the second most important reservoir (29.9%, 95% CI 0.5–91.8%) and cattle was the reservoir with the smallest amount of attributable cases (11.7%, 95% CI 0.3–40.8; **Table 4**).

**Table 4:** Attribution estimates obtained by the Asymmetric Island model provided with the complete datasets for *S*. Typhimurium and *S*. 4,[5],12:i:-, the merged dataset including all isolates available for both serovars and the bootstrap dataset.

	*S*. Typhimurium	*S*. 4,[5],12:i:-	*S*. Typhimurium + *S*. 4,[5],12:i:-	
			Merged dataset	Bootstrap dataset
Pig	64.4 (27.7–89.3)	58.4 (1.8–96.2)	67.0 (11.8–97.6)	87.7 (69.1–98.4)
Cattle	21.5 (0.5–63.8)	11.7 (0.3–40.8)	20.6 (0.3–80.7)	8.4 (0.3–25.4)
Poultry	14.1 (0.3–45.6)	29.9 (0.5–91.8)	12.4 (0.2–49.1)	3.9 (0.1–14.3)

For both serovars and for all sources, the attributions presented extremely large credibility intervals, leading to an excessive uncertainty, which hampered the robustness of the estimation model. As an attempt to improve the precision of the estimations, the sample size for each source was enlarged by merging the two original datasets into a unique dataset including all MLVA profiles associated with both *S.* Typhimurium and *S*. 4,[5],12:i:- isolates. With the merged dataset the estimations remained similar to the ones obtained with the *S*. Typhimurium dataset in terms of ranking of the different sources, and confidence intervals were still large, so that the source estimates still carried a large uncertainty (**Table 4**).

Since a possible bias of the source assignment could be the large difference in sample size among the putative sources investigated, the model was also run with a bootstrap dataset constructed sampling with replacement from each original source dataset and including 100 MLVA profiles per source. The bootstrap dataset provided the same ranking of sources as the merged dataset, but the relative importance of pigs as a source increased from 67.0% (95% CI 11.8–97.6) to 87.7% (95% CI 69.1–98.4), whereas for the other sources the attributions decreased. Moreover, the equal source size attribution led to a reduction of the uncertainty associate with the attribution estimates for all sources (**Table 4**).

#### Analysis of Molecular Variance

The AMOVA analysis was conducted on the merged dataset (including *S*. Typhimurium and *S*. 4,[5],12:i:- isolates). The results obtained are presented in **Table 5**. This analysis confirmed that almost the entire variance of the MLVA profiles (97.3%) was attributable to the within-source differences, whereas the proportion of the variance due to between-source differences was negligible (1.2%), as was the variance between human and non-human isolates (1.5%). The Phi statistics indicate that there were no significant population structural differences, thereby indicating that the sources did not contain significantly genetically differentiated MLVA profiles.

**Table 5:** Analysis of molecular variance (AMOVA), describing the population variation between human/non-human isolates, between isolates from different sources and within each isolate.

	Covariance components			
	Sigma	%	Φ-statistic (**p**-value)	
Between human – non-human	33.65	1.53	Φ_ST_	0.0153 (0.164)
Between different sources (human/pig/cattle/turkey/chicken)	26.59	1.21	Φ_CT_	0.0123 (0.516)
Within isolates	2143.80	97.27	Φ_SC_	0.0273
Total variations	2204.05	100.00		

## Discussion

The analysis of the MLVA profiles of *S*. Typhimurium and *S*. 4,[5],12:i:- isolates demonstrated that, in spite of the high similarity and close relationship between the two serovars, as previously described (Switt et al., 2009), and in spite of the considerable diversity of subtypes associated to both serovars, the heterogeneity of MLVA profiles of serovar *S*. 4,[5],12:i:- was more limited in comparison to *S*. Typhimurium.

This finding is in conformity with previous studies which compared the two serovars by using phenotypic methods (Barco et al., 2012), as well as molecular methods (Alcaine et al., 2006; Zamperini et al., 2007; Dionisi et al., 2009; Soyer et al., 2009) leading in all cases to the evidence that *S*. 4,[5],12:i:- variability is more limited than *S*. Typhimurium variability. This may indicate that *S*. 4,[5],12:i:- are recently emerged clones, and also that the genesis of the monophasic variants did not happen uniformly among the different clones of serovar Typhimurium.

In the present study, isolates were typed by using MLVA, which is classified as a highly discriminative subtyping method, since it targets highly unstable genetic markers (Chang et al., 2007). Hence, the identification of a discrete number of shared MLVA profiles between the two serovars (accounting for 59.40 and 73.97% of the isolates classified as *S*. Typhimurium and *S.* 4,[5],12:i:- respectively) strengthens the evidence that they are very closely related to each other. Cluster analysis subdivided the dataset into clusters peculiar for *S*. Typhimurium isolates and some other clusters including MLVA profiles displayed by both serovars. The high similarity between *S*. 4,[5],12:i:- and some *S*. Typhimurium isolates and the lower heterogeneity of the former serovar compared to the latter corroborate the hypothesis (Soyer et al., 2009; Hauser et al., 2010) that *S*. 4,[5],12:i:- may have evolved from a selection of recent *S*. Typhimurium ancestors.

Irrespective of the source of isolation, for *S*. 4,[5],12:i:- the MLVA discrimination was only associated to two loci (STTR5 and 6) out of the five loci investigated. The remaining loci were almost constantly absent (STTR10) or highly stable (STTR3 and 9). STTR5 and 6 were also the most polymorphic loci for *S.* Typhimurium. Laorden et al. (2010), who typed a collection of unrelated isolates of *S*. 4,[5],12:i:- using the same MLVA scheme, also observed that the discriminatory power was exclusively related to the diversity of STTR5 and 6. Other authors (Hopkins et al., 2010, 2012; Gallati et al., 2013; Garcia et al., 2013; Arguello et al., 2014; Boland et al., 2014), who characterized epidemiologically unrelated isolates of *S.* 4,[5],12:i:- by MLVA, reported profiles similar to those found in the present study. Since MLVA is a highly discriminatory method, Hopkins et al. (2007) reported that some minor changes in targeted loci could be tolerated among related outbreak isolates. Gain or loss of a single repeat unit and occasionally changes involving more repeat units in one of these highly variable loci (STTR5 and 6) have been described among epidemiological related *S.* 4,[5],12:i:- isolates obtained in the context of outbreak investigations by several authors (Petersen et al., 2011; Barco et al., 2013; Lettini et al., 2014). Hence, to interpret MLVA profiles, and as previously indicated for *Escherichia coli* O157:H7 (Noller et al., 2003), for *Salmonella,* a difference of one or two repeats at one single locus was also proposed (Hopkins et al., 2007; Petersen et al., 2011) as a cut-off to identify isolates that are part of the same outbreak. In this situation, where the standardized MLVA protocol for typing of *S*. 4,[5],12:i:- includes three highly stable loci, and two variable loci in which gain or loss of single repeat units is meaningless since such minor changes can be detected within related outbreak isolates, it is evident that interpretation of MLVA profiles can be challenging especially when the methodology is used to characterize temporally and geographically unrelated isolates. Hence, although MLVA has been depicted as one of the most promising subtyping methodologies to conduct large scale epidemiological studies for *Salmonella*, such as source attribution (Best et al., 2007; Barco et al., 2013; European Food Safety Authority [EFSA], 2013), the results of the present study pose questions on its applicability in this specific context.

The presence of identical or closely related MLVA profiles among isolates from different animal sources and humans reinforced the evidence that food-producing animals have an active involvement in the dissemination of *S*. Typhimurium and *S*. 4,[5],12:i:- through the human food chain. This finding was consistent with the results described by Best et al. (2007), who compared VNTR profiles from human and veterinary *S*. Typhimurium isolates (pig and poultry) and described a genetic overlap between VNTR profiles among the different species. However, when we tried to rank the species in terms of their importance as sources of human infections the picture obtained was complicated. Although isolates from humans, showed for both serovars, some overlaps with isolates from different species, the populations of MLVA profiles produced were not clearly structured such that the host could be easily inferred from the genotypes. For *S*. 4,[5],12:i:-, a consistent genetic overlap was noted among human isolates and isolates from all species suggesting multiple contamination sources. For *S*. Typhimurium, in contrast very few MLVA profiles were shared between human and non-human isolates, even though the majority of human isolates showed high genetic similarities with isolates from different sources. This divergence between the two serovars can be related to the different level of clonality associated with the two serovars, as previously discussed.

The high discriminatory power of molecular subtyping methods makes source attribution difficult if sources are attributed simply based on the exact overlap of subtypes. Hence, population genetic models, taking into account the genetic relationship among isolates (based on analysis of mutations, recombination, and migrations), have been identified as valuable tools to further clarify the relevant host associations and to identify the key reservoirs when molecular subtyping data are available (Mullner et al., 2009; Mughini-Gras et al., 2014a).

In the present study, the Asymmetric Island Model supplied with the MLVA profiles was used to infer sources of human infections. For both *S*. Typhimurium and *S.* 4,[5],12:i:-, the model attributed the majority of human cases to pig, confirming the results previously obtained by using frequency-matched models to attribute the source of human salmonellosis in Italy. In particular, pig was identified as the major source of human salmonellosis (considering all serovars) in Italy by using the Dutch and modified Hald source attribution models supplied with serotyping data collected at the national level over the period 2002–2010 (Mughini-Gras et al., 2014b). This conclusion was consistent with that previously obtained by using similar approaches to analyze different datasets (Pires et al., 2011). Differently from these findings, poultry is described as the main source of human salmonellosis in the majority of the European countries (Pires et al., 2011), and in the United States (Chen and Jiang, 2014).

These differences between countries in the relative contribution of different food sources to human salmonellosis can be explained by several factors, such as the differences in animal and food production systems, the food consumption and preparation habits, the epidemiology of the pathogen and the efficiency of surveillance programs in place in different regions (Pires et al., 2011).

Nevertheless, for both serovars and for all sources investigated the attributions provided by the Asymmetric Island Model presented large credibility intervals, leading to an excessive uncertainty, which hampered the robustness of the estimations. Unfortunately, merging the two original datasets into a unique dataset did not produce a substantial constriction of the credibility intervals. Smid et al. (2013), who used the Asymmetric Island Model to identify the sources of human campylobacteriosis, concluded that it is advisable to have over 100 isolates per food source to perform source attribution studies using the model and to obtain a satisfactory statistical power. To confirm this hypothesis, the model was also run with a bootstrap dataset including 100 MLVA profiles per source, and this exercise led to a reduction of the uncertainty associated with the attribution estimates. The need for a substantial dataset to get reliable estimations by the Asymmetric Island Model was also demonstrated by Mughini-Gras et al. (2014a). These authors described reliable estimations about sources of human salmonellosis due to *S*. Typhimurium-*S*. 4,[5],12:i:- and *S*. Enteritidis in The Netherlands by using the model provided with large datasets of MLVA data.

Therefore, it seems relevant to enlarge the available datasets, so that even for the more rare sources at least 100 epidemiologically independent isolates can be available. Another important issue to take into account when putative sources of infection are inferred by using molecular data is the genetic differentiation between groups (sources), which must be higher than the within group heterogeneity in order to get robust estimations. In particular, in the case of a noteworthy heterogeneity within each source and a weak genetic differentiation among sources the degree of accuracy in the source assignments can be jeopardized (Wilson et al., 2008). When the AMOVA was used to quantify the genetic differentiation within and between the different sources investigated in the present study this prerequisite was shown not to be fulfilled, i.e., there was high heterogeneity found within each source and negligible divergence between sources.

Source attribution studies rely on subtyping methods which should have enough discriminatory power to identify links between human isolates and their putative sources, but they should not be too discriminatory, so that true epidemiological association between isolates might be missed. The current 5-loci MLVA scheme does not seem to fulfill this requirement, particularly for *S*. 4,[5],12:i:-. Although MLVA has often been presented as one of the most promising subtyping methodologies to support outbreak investigations, the results of the present study pose significant questions about its effective applicability for conducting large scale epidemiological studies such as source attribution. The *S.* Typhimurium 5-loci MLVA scheme, especially when used to type *S.* 4,[5],12:i:- isolates, showed three stable and two highly variable loci. Hence, MLVA provides fingerprinting of a narrow and highly variable tract of the DNA, thereby complicating the characterization, especially for epidemiologically unrelated isolates.

## Author Contributions

Conceived and designed the experiments: LB, FB, JO, IL, AL, AR. Performed the experiments: FB, EC, ER, AL. Analyzed the data: LB, FB, EC, ER, AL, AR. Contributed strains: IL. Critical discussion about the results: LB, FB, EC, ER, JO, IL, AL, AR. Wrote the paper: LB, FB, AL, JO, AR. Final approval for the submitted document: LB, FB, EC, ER, JO, IL, AL, AR.

## Conflict of Interest Statement

The authors declare that the research was conducted in the absence of any commercial or financial relationships that could be construed as a potential conflict of interest.

## References

[B1] Alcaine S. D. Soyer Y. Warnick L. D. Su W. L. Sukhnanand S. Richards J. (2006). Multilocus sequence typing supports the hypothesis that cow- and human- associated *Salmonella* isolates represent distinct and overlapping populations. *Appl. Environ. Microbiol.* 72 7575–7585 10.1128/AEM.01174-06 17028236 PMC1694263

[B2] Arguello H. Sorensen G. Carvajal A. Baggesen D. L. Rubio P. Pedersen K. (2014). Characterization of the emerging *Salmonella* 4,[5],12.i:- in Danish animal production. *Foodborne Pathog. Dis.* 11 366–372 10.1089/fpd.2013.1672 24673107

[B3] Barco L. Barrucci F. Olsen J. E. Ricci A. (2013). *Salmonella* source attribution based on microbial subtyping. *Int. J. Food Microbiol.* 163 193–203 10.1016/j.ijfoodmicro.2013.03.005 23562696

[B4] Barco L. Lettini A. A. Ramon E. Longo A. Saccardin C. Dalla Pozza M. C. (2011). A rapid and sensitive method to identify and differentiate *Salmonella enterica* serotype Typhimurium and *Salmonella enterica* serotype 4,[5],12:i:- by combining traditional serotyping and multiplex polymerase chain reaction. *Foodborne Pathog. Dis.* 8 741–743 10.1089/fpd.2010.0776 21247297 PMC3117292

[B5] Barco L. Mancin M. Ruffa M. Saccardin C. Minorello C. Zavagnin P. (2012). Application of the random forest method to analyse epidemiological and phenotypic characteristics of *Salmonella* 4,[5],12:i:- and *Salmonella* Typhimurium strains. *Zoonoses Public Health* 59 505–512 10.1111/j.1863-2378.2012.01487.x 22583909

[B6] Best E. L. Lindstedt B. A. Cook A. Hadley F. A. C. Threlfall E. J. Liebana E. (2007). Multiple-locus variable-number tandem repeat analysis of *Salmonella enterica* subsp. *enterica* serovar Typhimurium: comparison of isolates from pigs, poultry and cases of human gastroenteritis. *J. Appl. Microbiol.* 103 565–572 10.1111/j.1365-2672.2007.03278.x 17714389

[B7] Boland C. Bertrand S. Mattheus W. Dierick K. Wattiau P. (2014). Molecular typing of monophasic *Salmonella* 4,[5]:i:- strains isolated in Belgium (2008-2011). *Vet. Microbiol.* 168 447–450 10.1016/j.vetmic.2013.11.040 24398228

[B8] Chang C. H. Chang Y. C. Underwood A. Chiou C. S. Kao C. Y. (2007). VNTRDB: a bacterial variable number tandem repeat locus database. *Nucleic Acids Res.* 35 D416–D421 10.1093/nar/gkl872 17175529 PMC1781188

[B9] Chen Z. Jiang X. (2014). Microbiological safety of chicken litter or chicken litter-based organic fertilizers: a review. *Agriculture* 4 1–29 10.3390/agriculture4010001

[B10] Dionisi A. M. Graziani C. Lucarelli C. Filetici E. Villa L. Owczarek S. (2009). Molecular characterization of multidrug-resistant strains of *Salmonella enterica* serotype Typhimurium and monophasic variant (S. 4[5],12:i) isolated from human infections in Italy. *Foodborne Pathog. Dis.* 6 711–717 10.1089/fpd.2008.0240 19580448

[B11] Dray S. Dufour A. B. (2007). The ade4 package: implementing the duality diagram for ecologists. *J. Stat. Softw.* 22 1–20.

[B12] Excoffier L. Smouse P. E. Quattro J. M. (1992). Analysis of molecular variance inferred from metric distances among DNA haplotypes: application to human mitochondrial DNA restriction data. *Genetics* 131 479–491. 1644282 10.1093/genetics/131.2.479 PMC1205020

[B13] European Food Safety Authority [EFSA]. (2008). Scientific opinion of the panel on biological Hazards on overview of methods for source attribution for human illness from food-borne microbiological hazards. *EFSA J.* 764 1–43. 10.2903/j.efsa.2008.764 PMC10193638 37213830

[B14] European Food Safety Authority [EFSA]. (2010). Scientific opinion of the panel on biological Hazards on monitoring and assessment of the public health risk of *Salmonella* Typhimurium-like strains. *EFSA J.* 8 1826[48pp].

[B15] European Food Safety Authority [EFSA]. (2013). Scientific opinion of the panel on biological hazards on the evaluation of molecular typing methods for major food-borne microbiological hazards and their use for attribution modeling, outbreak investigation and scanning surveillance: part 1 (evaluation of methods and applications). *EFSA J.* 11 3502[84pp].

[B16] European Food Safety Authority [EFSA], and European Centre for Disease Prevention, and Control [ECDC]. (2014). The European Union summary report on trends and sources of zoonoses, zoonotic agents and food-borne outbreaks in 2012. *EFSA J.* 12 3547[312pp]. 10.2903/j.efsa.2014.3547 PMC7009540 32625785

[B17] Gallati C. Stephan R. Hacler H. Malorny B. Schroeter A. Nuesch-Inderbinen M. (2013). Characterization of *Salmonella enterica* subsp. *enterica* serovar 4,[5],12:i:- clones isolated from human and other sources in Switzerland between 2007 and 2011. *Foodborne Pathog. Dis.* 10 549–554 10.1089/fpd.2012.1407 23614800

[B18] Garcia P. Malorny B. Hauser E. Mendoza M. C. Rodicio M. R. (2013). Genetic types, gene repertoire, and evolution of isolates of the *Salmonella enterica* serovar 4,5,12:i:- Spanish clone assigned to different phage types. *J. Clin. Microbiol.* 51 973–978 10.1128/JCM.02777-12 23325816 PMC3592032

[B19] Graziani C. Mughini-Gras L. Owczarek S. Dionisi A. Luzzi I. Busani L. (2013). Distribution of *Salmonella enterica* isolates from human cases in Italy, 1980-2011. *Euro Surveill.* 18. 23870078

[B20] Grimont P. A. D. Weill F.-X. (2007). *Antigenic Formulae of Salmonella Serovars* 9th Edn. Paris: WHO Collaborating Centre for Reference and Research on *Salmonella*. Institut Pasteur.

[B21] Hauser E. Tietze E. Helmuth R. Junker E. Blank K. Prager R. (2010). Pork contamination with *Salmonella enterica* serovar 4,[5],12:i:-, an emerging health risk for humans. *Appl. Environ. Microbiol.* 76 4601–4610 10.1128/AEM.02991-09 20472721 PMC2901716

[B22] Havelaar A. H. Brauning J. Christiansen K. Cornu M. Hald T. Mangen M. J. (2007). Towards an integrated approach in supporting microbial food safety decisions. *Zoonoses Public Health* 54 103–117 10.1111/j.1863-2378.2007.01036.x 17456140

[B23] Heck M. (2009). Multilocus variable number of tandem repeats analysis (MLVA) – a reliable tool for rapid investigation of *Salmonella* Typhimurium outbreaks. *Euro surveill.* 14. 19371517

[B24] Hopkins K. L. de Pinna E. Wain J. (2012). Prevalence of *Salmonella enterica* serovar 4[5],12:i:- in England and Wales, 2010. *Euro surveill.* 17. 22995432

[B25] Hopkins K. L. Kirchner M. Guerra B. Granier S. A. Lucarelli C. Porrero M. C. (2010). Multiresistant *Salmonella enterica* serovar 4[5],12:i:- in Europe: a new pandemic strain? *Euro Surveill.* 15 19580. 20546690

[B26] Hopkins K. L. Maguire C. Best E. Liebana E. Threlfall E. J. (2007). Stability of multiple-locus variable number tandem repeats in *Salmonella enterica* serovar Typhimurium. *J. Clin. Microbiol.* 45 3058–3061 10.1128/JCM.00715-07 17609320 PMC2045244

[B27] Laorden L. Herrera-Leon S. Martinez I. Sanchez A. Kromidas L. Bikandi J. (2010). Genetic evolution of Spanish multidrug-resistant *Salmonella enterica* 4,5,12:i:- monophasic variant. *J. Clin. Microbiol.* 48 4563–4566 10.1128/JCM.00337-10 20943866 PMC3008487

[B28] Larsson J. T. Torpdahl M. MLVA Working Group Møller N. E. (2013). Proof-of-concept study for successful inter-laboratory comparison of MLVA results. *Euro Surveill.* 18 20566 10.2807/1560-7917.ES2013.18.35.20566 24008232

[B29] Larsson J. Torpdahl M. Petersen R. F. Sorensen G. Lindstedt B. A. Nielsen E. M. (2009). Development of a new nomenclature for *Salmonella* Typhimurium multi-locus tandem repeats analysis (MLVA). *Euro Surveill.* 14. 19371515

[B30] Lettini A. A. Saccardin C. Ramon E. Longo A. Cortini E. Dalla Pozza M. C. (2014). Characterization of an unusual *Salmonella* phage type DT7a and report of a foodborne outbreak of Salmonellosis. *Int. J. Food Microbiol.* 189 11–17 10.1016/j.ijfoodmicro.2014.07.021 25108760

[B31] Lindstedt B. A. Vardund T. Aas L. Kapperud G. (2004). Multiple-locus variable-number tandem-repeats analysis of *Salmonella enterica* subsp. *enterica* serovar Typhimurium using PCR multiplexing and multicolor capillary electrophoresis. *J. Microbiol. Methods* 59 163–172 10.1016/j.mimet.2004.06.014 15369852

[B32] Mughini-Gras L. Smid J. Enserink R. Franz E. Schouls L. Heck M. (2014a). Tracing the sources of human salmonellosis: a multi-model comparison of phenotyping and genotyping methods. *Infect. Genet. Evol.* 28 251–260 10.1016/j.meegid.2014.10.003 25315490

[B33] Mughini-Gras L. Barrucci F. Smid J. H. Graziani C. Luzzi I. Ricci A. (2014b). Attribution of human *Salmonella* infections to animal and food sources in Italy (2002-2010): adaptations of the Dutch and modified Hald source attribution models. *Epidemiol. Infect.* 142 1070–1082 10.1017/S0950268813001829 23920400 PMC9151150

[B34] Mullner P. Spencer S. E. F. Wilson D. J. Jones G. Noble A. D. Midwinter A. C. (2009). Assigning the source of human campylobacteriosis in New Zeland: a comparative genetic and epidemiological approach. *Infect. Genet. Evol.* 9 1311–1319 10.1016/j.meegid.2009.09.003 19778636

[B35] Noller A. C. McEllistrem M. C. Pacheco A. G. F. Boxrud D. J. Harrison H. L. (2003). Multilocus variable-number tandem repeat analysis distinguishes outbreak and sporadic *Escherichia coli* O157:H7 isolates. *J. Clin. Microbiol.* 41 5389–5397 10.1128/JCM.41.12.5389-5397.2003 14662916 PMC308975

[B36] Petersen R. F. Litrup E. Larsson J. T. Torpdahl M. Sorensen G. Muller L. (2011). Molecular cheracterization of *Salmonella* Typhimurium highly successful outbreak strains. *Foodborne Pathog. Dis.* 8 655–661 10.1089/fpd.2010.0683 21381921

[B37] Pires S.M. de Knegt L. Hald T. (2011). *Estimation of the* *Relative Contribution of Different Food and Animal Sources to Human Salmonella Infections in the European Union* Scientific/Technical Report submitted to EFSA. National Food Institute, Technical University of Denmark, Kongens Lyngby. Available at: http://www.efsa.europa.eu/it/supporting/doc/184e.pdf

[B38] Pires S. M. Evers E. G. van Pelt W. Ayers T. Scallan E. Angulo F. J. (2009). Attributing the human disease burden of foodborne infections to specific sources. *Foodborne Pathog. Dis.* 6 417–424 10.1089/fpd.2008.0208 19415971

[B39] R Core Team. (2012). *R: A Language and Environment for Statistical Computing.* Vienna: R Foundation for Statistical Computing. Available at: http://www.R-project.org/

[B40] Ross I. L. Davos D. E. Mwanri L. Raupach J. Heuzenroeder M. W. (2011). MLVA and phage typing as complementary tools in the epidemiological investigation of *Salmonella enterica* serovar Typhimurium clusters. *Curr. Microbiol.* 62 1034–1038 10.1007/s00284-010-9820-1 21104081

[B41] Smid J. H. Mughini-Gras L. de Boer A. G. French N. P. Havelaar A. H. Wagenaar J. A. (2013). Practicalities of using non-local or non-recent multilocus sequence typing data for source attribution in space and time of human campylobacteriosis. *PLoS ONE* 8:e55029 10.1371/journal.pone.0055029 PMC3566096 23405107

[B42] Soyer Y. Switt A. M. Davis M. A. Maurer J. McDonough P. L. Schoonmaker-Bopp D. J. (2009). Salmonella enterica serotype 4,5,12:i:-, an emerging serotype that represents multiple distinct clones. *J. Clin. Microbiol.* 47 3546–3556 10.1128/JCM.00546-09 19741087 PMC2772592

[B43] Switt A. L. Soyer Y. Warnick L. D. Wiedmann M. (2009). Emergence, distribution, and molecular and phenotypic characteristics of *Salmonella enterica* serotype 4,5,12:i:-. *Foodborne Pathog. Dis.* 6 407–415 10.1089/fpd.2008.0213 19292687 PMC3186709

[B44] Torpdahl M. Sorensen G. Lindstedt B. A. Nielsen E. M. (2007). Tandem repeat analysis for surveillance of human *Salmonella* Typhimurium infections. *Emerg. Infect. Dis.* 13 388–395 10.3201/eid1303.060460 17552091 PMC2725892

[B45] Wilson D. J. Gabriel E. Leatherbarrow A. J. Cheesbrough J. Gee S. Bolton E. (2008). Tracing the source of campylobacteriosis. *PLoS Genet.* 4:e1000203 10.1371/journal.pgen.1000203 PMC2538567 18818764

[B46] Wuyts V. Mattheus W. De Laminne de Bex G. Wildemauwe C. Roosens N. H. (2013). MLVA as tool for public health surveillance of human *Salmonella* Typhimurium: prospective study in Belgium and evaluation of MLVA loci stability. *PLoS ONE* 8:e84055 10.1371/journal.pone.0084055 PMC3877154 24391880

[B47] Zamperini K. Soni V. Waltman D. Sanchez S. Theriault E. C. Bray J. (2007). Molecular characterization reveals *Salmonella enterica* serovar 4,[5],12:i:- from poultry is a variant Typhimurium serovar. *Avian Dis.* 51 958–964 10.1637/7944-021507-REGR.1 18251408

